# A Simple Drain Current Model for MOS Transistors with the Lorentz Force Effect

**DOI:** 10.3390/s17061199

**Published:** 2017-05-24

**Authors:** Hwang-Cherng Chow, Prasenjit Chatterjee, Wu-Shiung Feng

**Affiliations:** Graduate Institute of Electronic Engineering, Chang Gung University, 259 Wenhwa 1st Road, Kweishan, Taoyuan 333, Taiwan; fengws@mail.cgu.edu.tw

**Keywords:** Lorentz force, Hall effect, MagFET, MOSFET, magnetic sensor

## Abstract

A novel concept of drain current modelling in rectangular normal MOS transistors with the Lorentz force has been proposed for the first time. The single-drain MOS transistor is qualified as a magnetic sensor. To create the Lorentz force, a DC loop current is applied through an on-chip metal loop around the device, and the relation between the applied loop current and the created magnetic field is assumed to be linear in nature. The drain current of the MOS transistor is reduced with the applied Lorentz force from both directions. This change in the drain current is ascribed to a change in mobility in the strong inversion region, and a change in mobility of around 4.45% is observed. To model this change, a set of novel drain current equations, under the Lorentz force, for the strong inversion region has been proposed. A satisfactory agreement of an average error of less than 2% between the measured and the calculated drain currents under the magnetic field created by an on-chip metal loop is achieved.

## 1. Introduction

In the past few decades, magnetic sensors have aided humans to analyse and control many functions due to their high stability [1,2,3]. Semiconductor-based magnetic sensors such as Hall sensors, magnetic field effect transistors (MagFET), magnetotransistors etc. are also very popular for detecting low-to-moderate magnetic fields and they can be produced at low cost. Although cheap and easy to produce, Hall sensors, especially when integrated inside real integrated circuits, are generally affected by the magnetic flux from the surroundings that may affect the field which Hall sensors intend to detect. To overcome this issue, the size of the device in other semiconductor-based sensors such as MagFETs and rectangular MOS transistors must be large enough so that the effect of the Hall voltage is suppressed. On the other hand, MagFETs sense the current imbalance between two drains due to the effect of the Lorentz force, but they also suffer from a large offset due to a mismatch between two drains, and they also suffer from temperature drift and noise [4,5]. Recently, rectangular normal MOS transistors have also been examined as magnetic sensors. In [6], a rectangular MOSFET with a special magnetic gate was reported, but the applied magnetic field was quite high and externally applied, and the sensitivity was also very poor. In [7], a normal-gate single drain MOSFET was reported with an on-chip magnetic field. The large aspect ratio of the device ensures the suppression of the Hall voltage to maximize sensitivity by enhancing magnetoresistance. In [7], the applied magnetic field was low while the sensitivity was higher than reported in the prior arts. However, in all the reported cases there is no model for the drain current using the Lorentz force to demonstrate the magnetoresistance effect [8].

In [9,10,11,12,13], several analytical or mathematical models have been proposed, but this approach of modelling for split-drain MagFET structures is very complicated. In [9], the device size is very big, and in [10,11], the analysis is done at very low temperatures impractical for real-life applications. Moreover, all of these prior arts [9,10,11,12,13] dealt with MagFET structures only. Therefore, there is no simple model for an MOS transistor as the magnetic sensor with Lorentz force effect, and these presented current models are difficult to use or to be integrated in circuit simulators.

In this paper it is shown that under the Lorentz force the change in the drain current is attributed to a change in mobility only in strong inversion. For the first time as far as we know, based on the change in mobility in the strong inversion region due to the applied on-chip magnetic field, a set of novel, empirical yet simple modified drain current equations for single-drain rectangular normal MOSFET has been proposed to validate the magnetoresistance effect.

## 2. Device Structure and Principle of Operations

**Figure 1:**
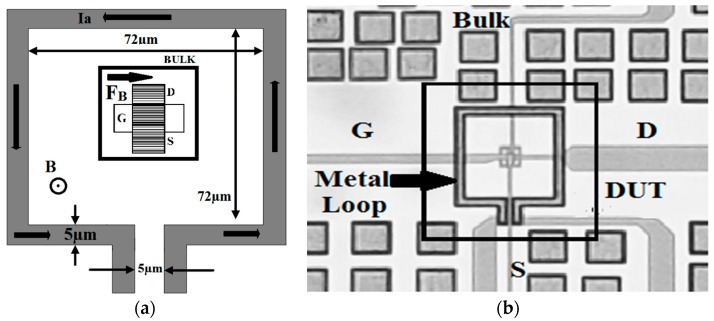
(**a**) Conceptual diagram of the device under test; (**b**) Chip Microphotograph.

A rectangular n-channel MOSFET is fabricated by using TSMC standard 0.18 μm CMOS technology with a channel length of 0.18 µm and a channel width of 18 µm. The gate oxide thickness is about 37 Å, and the gate material is polysilicon. Figure 1a represents the conceptual test device diagram with a metal loop, and the black square in Figure 1b represents the device under test (DUT).

A square-shaped metal loop 72 µm long on each side and 5 µm wide is placed around the device to create both a steady and uniform magnetic field (B) around the active region of the device. To create the magnetic field, a DC current is passed through the metal loop from 0 mA to 100 mA with steps of 20 mA. However, a current of 100 mA is very high for a width of 5 µm and can produce heat in the loop and on the surface of the DUT. To overcome this issue, a 5 W 100 Ω external ceramic resistor, connected in series with the metal loop, is used during measurements. This resistor takes care of the heating issue outside of the chip surface. Moreover, using the automatic test equipment, the time interval of applying the loop current is very short and hence the chip surface temperature is not changed significantly ensuring literally no damage to the metal loop as well as to the sensing device. Furthermore, the MOSFET used here is the sensing device. This metal loop is only used to create an on-chip magnetic field to demonstrate the magnetic sensing function of the MOSFET. Therefore, when the sensing device operates in real-life high-density circuits, there will be only a very limited amount of dissipated heat (as in the case of a normal MOSFET) since the demonstrated metal loop is not required.

Figure 1b represents the fabricated chip microphotograph where the terminals of the transistor are placed differently than in Figure 1a for ease of probing the device. The measurements are carried out by an Agilent B1500A semiconductor parameter analyser in an anti-vibration probe station, as mentioned in [7]. In MOSFETs with a large W/L ratio (W/L > 5), the Hall Effect is effectively suppressed which leads to a maximum magnetoresistance effect, resulting only from the effect of Lorentz force [14]. In other words, with a lower W/L ratio the device behavior will be affected by the Hall voltage along with the Lorentz force, and the maximum change in mobility of the charged particles is not possible, leading to a drop in sensitivity of the magnetic sensor. Many test devices with different W/L ratios, (W/L > 5), have been fabricated, and for those devices the results have so far been consistent. The Lorentz force F_B_ in Figure 1a is created by applying the loop current as mentioned earlier. This loop current has a linear relationship with the created magnetic field. Hence, loop current and magnetic field are synonymous throughout this article unless otherwise specified. Due to both the loop current and the moving particles, this Lorentz will change the effective magnetoresistance of the carriers [8,14], which in turn reduces the mobility of the carriers in the strong inversion region of the device. In the strong inversion region of operation, the drift current dominates and hence the velocity of the carrier is high. Under these circumstances, the applied Lorentz force changes the effective magnetoresistance of the carriers and the mobility is reduced which in turn also reduces the drain current.

## 3. Experimental Results and Discussion

**Figure 2:**
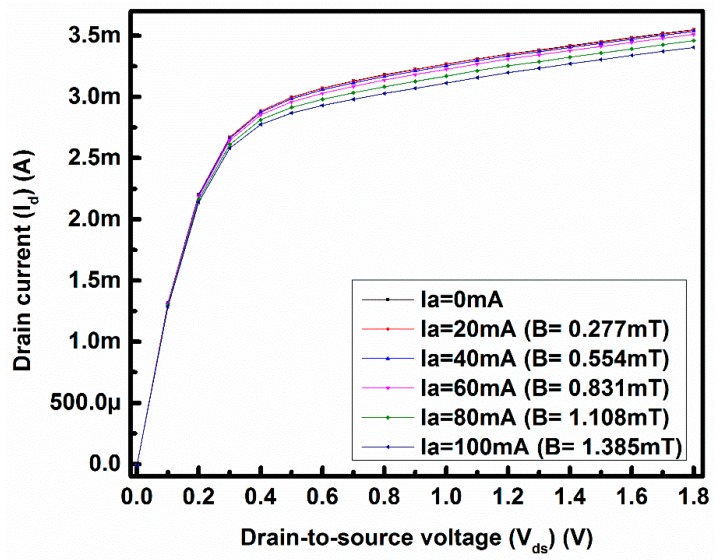
Measured I_d_-V_ds_ at fixed V_gs_ = 1 V under different I_a_ ranging from 0 mA to 100 mA.

Here, a rectangular-shaped MOSFET is used as a magnetic sensor. The magnetic field (B) is created by applying a DC current (I_a_) through the metal loop from 0 mA to 100 mA with steps of 20 mA, as stated above. The direction of the applied magnetic field is the same as mentioned in [7]. The metal loop, used as a magnetic field generator, is placed in proximity to the devices to ensure a uniform magnetic field around the active region of the devices and to minimize the spatial variations. The estimated strength of the magnetic field with the 100 mA loop current is 1.385 mT, and the values of the strength of the magnetic field for other loop currents are provided in Figure 2. This applied magnetic field generates the Lorentz force that ultimately decreases the drain current of the sensing MOSFET by altering magnetoresistance. The DUT is biased in the strong inversion region to understand the behavior of the charged particles under the applied magnetic field.

To understand the effect of an on-chip low magnetic field on the device characteristics, the drain current (I_d_) is measured with respect to the drain-to-source voltage (V_ds_) where the gate-to-source voltage (V_gs_) is fixed at 1 V to ensure that the device is operating in the strong inversion region of operation. Figure 2 represents the I_d_-V_ds_ curve for a fixed V_gs_, where the horizontal axis represents the drain-to-source voltage V_ds_ and the vertical axis represents the drain current I_d_. From Figure 2 it can be seen, that the measured drain current decreases with increasing loop current (I_a_). As the loop current increases, the Lorentz force is also increases, which changes the magnetoresistance of the charged particles. As a result, the drain current decreases. This change in I_d_ can be only referred to a change in carrier mobility by the magnetroresistance effect. Due to the direction of the applied magnetic field, the threshold voltage is assumed as without change with the Lorentz force effect.

On the other hand, the mobility of the charged carriers can be affected by various scattering factors such as mobility related to scattering by bulk and surface phonons, screen Coulomb scattering, surface roughness scattering etc. Under the Lorentz force, these scattering factors become more dominant, which in turn decreases the drain current by reducing the effective mobility of the charged carriers.

**Figure 3:**
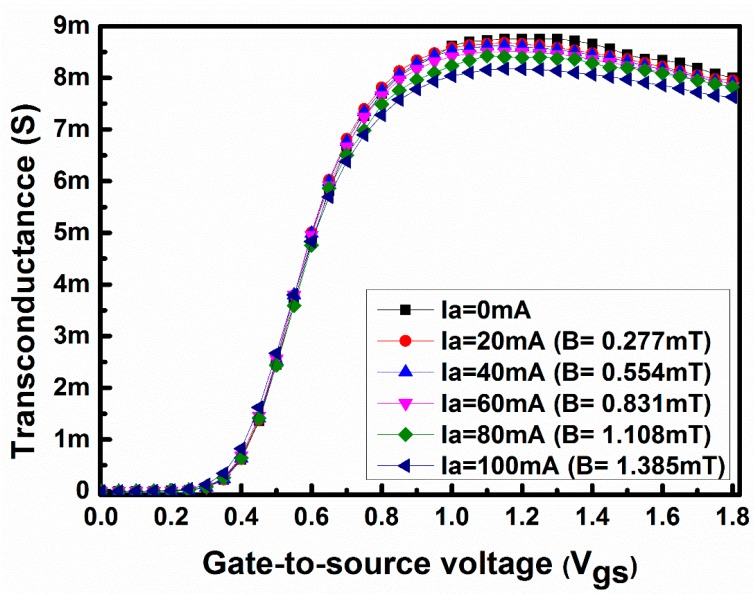
Transconductance with V_gs_ for different applied loop currents at V_ds_ = 1.8 V.

To ensure that the change in mobility is solely due to the change in magnetoresistance of the charged particles, the transconductance is plotted against the gate-to-source voltage based on the measured I_d_-V_gs_ data in Figure 3. The decrease of the drain current in Figure 2 and the decrease in transconductance in Figure 3 with the applied magnetic field reveal the clear magnetoresistance effect [8,14].

From the discussions follows that under the influence of the Lorentz force the normal MOS transistor biased in the strong inversion region of operation exhibits a change in magnetoresistance of the charged carriers, which causes the change in mobility. This change in mobility might be modelled using a set of novel modified drain current equations for MOS transistors under a steady low magnetic field. The modified drain current equations are as follows:

(1) $$I_{d}=\;\frac{1}{2}\;\left(\mu_{\mathrm{eff}}\right)\frac{\varepsilon_{\mathrm{ox}}}{t_{\mathrm{ox}}}\frac{W}{L}\;{\left[V_{\mathrm{gs}}-V_{t}\right]}^{2}\left(1+{\lambda \;V}_{\mathrm{ds}}\right)$$

(2) $$I_{d}=\;\left(\mu_{\mathrm{eff}}\right)\frac{\varepsilon_{\mathrm{ox}}}{t_{\mathrm{ox}}}\frac{W}{L}\;\left[\left[V_{\mathrm{gs}}-V_{t}\right]V_{\mathrm{ds}}-\frac{1}{2}V_{\mathrm{ds}}^{2}\right]\left(1+{\lambda \;V}_{\mathrm{ds}}\right)$$

(3) $$\mathrm{where}{\;\mu }_{\mathrm{eff}}=\;\left[\frac{\mu_{0}}{1+\;\theta \;\left(V_{\mathrm{gs}}-V_{t}\right)}\right]-{\alpha I}_{a}^{2}$$

(4) $$V_{t}={\;V}_{t0}-{\;\eta V}_{\mathrm{ds}}$$

In Equations (1)–(4), λ is the channel length modulation parameter, $\varepsilon_{\mathrm{ox}}$ is the permittivity of the silicon di-oxide, $t_{\mathrm{ox}\;}\;$ is the thickness of the silicon di-oxide, η is the drain-induced barrier lowering (DIBL) parameter and θ is a fitting parameter for mobility calculation. The value of λ for the channel length modulation is extracted from the slope of the measured values of the drain current, and the average extracted value of λ is 0.0849 [7]. The value of the threshold voltage (V_t_) is affected by the DIBL effect, and the extracted value of the V_t0_ is 0.31 V and the extracted value of η in Equation (4) is 0.01. On the other hand, α is a fitting parameter whose value can be calculated from the change in mobility due to the Lorentz effect. This parameter α is introduced to consider the effects of both the geometrical correction factor and the Lorentz force. To calculate the value of α, the mobility of the device is calculated considering the effect of η, and the maximum extracted value of mobility with DIBL effect is 137.67 cm^2^ V^−1^ s^−1^.

**Figure 4:**
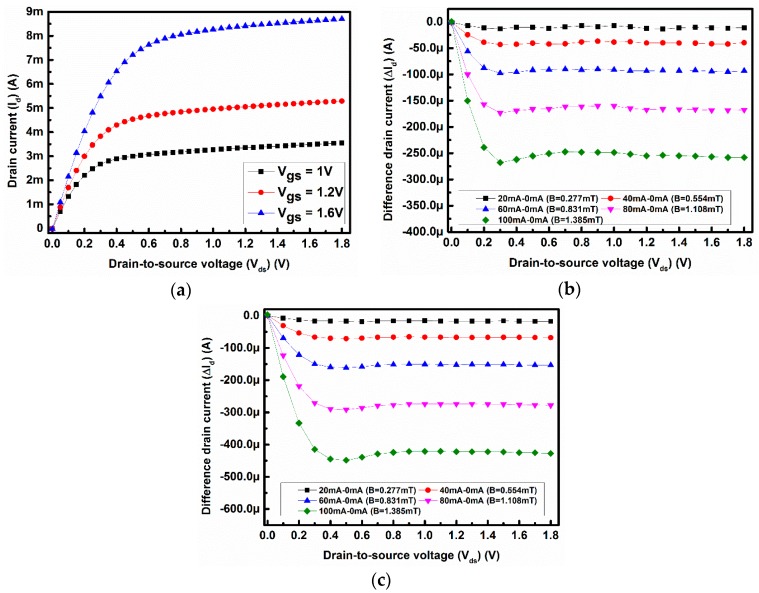
(**a**) Measured drain current with drain-to-source voltage at V_gs_ = 1 V, V_gs_ = 1.2 V and V_gs_ = 1.6 V with no loop current (B = 0); (**b**) difference drain current with drain-to-source voltage at V_gs_ = 1.2 V (B = 0–1.385 mT); (**c**) difference drain current with drain-to-source voltage at V_gs_ = 1.6 V (B = 0–1.385 mT).

Figure 4a shows the measured values of the drain current of the MOSFET sensor with the drain-to -source voltage when no magnetic field is applied. Both Figure 4b,c represent the difference in drain current with drain-to-source voltage and gate-to-source voltage of 1.2 V and 1.6 V, respectively. The on-chip magnetic field is created by applying a DC current through the metal loop, as mentioned earlier. The difference in drain current is calculated by subtracting the drain current with the applied magnetic field from the drain current without the applied magnetic field. From Figure 4b to Figure 4c it is evident that as the strength of the magnetic field (i.e., applied current through the metal loop) increases the difference current also increases due to a stronger Lorentz force F_B_, as shown in Figure 1a.

**Figure 5:**
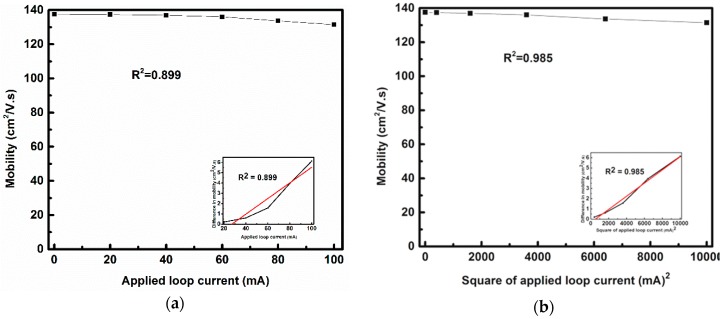
(**a**) Change in mobility with loop current; (**b**) Change in mobility with squared loop current.

**Figure 6:**
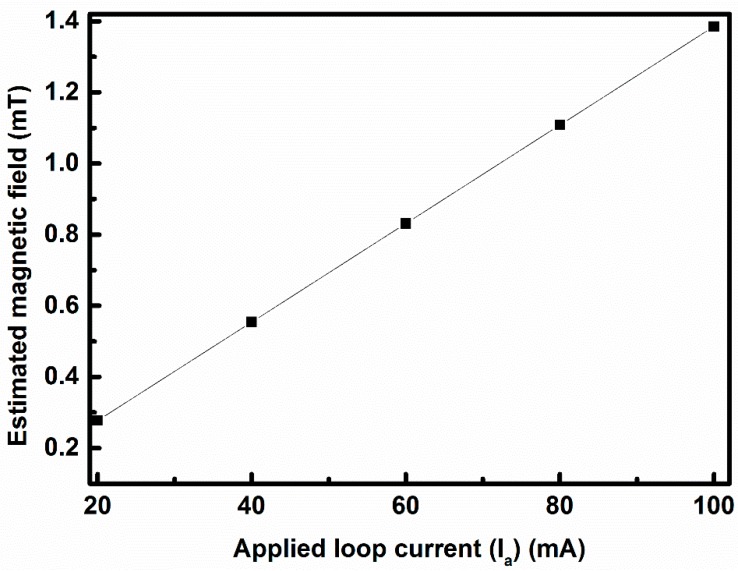
Estimated magnetic field with the applied loop current.

Figure 5a represents the change in mobility with the loop current, while the change in mobility shows better linearity with the squared loop current as shown in Figure 5b since the value of fitting linearity can be improved from 0.899 in Figure 5a to 0.985 in Figure 5b. Based on ideal theoretical analysis, the generated field strength of B is linearly proportional to the loop current only. As shown in Figure 6, the applied magnetic field has a linear relationship with the applied loop current, i.e., both the applied loop current and the applied magnetic field are synonymous in this case. From this change in mobility due to the Lorentz force, the value of α is calculated, and the average value of α is 5.094 × 10^−4^ cm^2^/(V s mA^2^). The value of α changes slightly with V_g_. However, based on our calibrations, this change is very small and for the sake of simplicity the value of α is considered constant for changing V_g_. Using Equation (3) and the value of $\mu_{\mathrm{eff}}$ for no loop current, the value of θ is obtained as 0.62 V^−1^. This value of θ is not effected by the change in Lorentz force. The value of mobility with a loop current of 100 mA is 131.54 cm^2^ V^−1^ s^−1^. A change of around 4.45% in mobility is observed.

The change in drain current due to the Lorentz effect can be written from Equation (1)

(5) $$\Delta I_{d}=\;\left|I_{d100}-{\;I}_{d0}\right|=\;\left|\frac{1}{2}\;\frac{\varepsilon_{\mathrm{ox}}}{t_{\mathrm{ox}}}\frac{W}{L}\;{\left[V_{\mathrm{gs}}-V_{t}\right]}^{2}\left(1+{\lambda \;V}_{\mathrm{ds}}\right)\;\left[\mu_{\mathrm{eff}100}-\mu_{\mathrm{eff}0}\right]\right|$$

where $I_{d100}$ and $I_{d0}$ are drain currents at 100 mA and 0 mA loop currents (I_a_), respectively, and $\mu_{\mathrm{eff}100}\;$and $\mu_{\mathrm{eff}0}$ are the respective mobility. Equation (5) can be re-written as

(6) ΔId=|Id100−Id0|=|Id0.[μeff100−μeff0]μeff0|

(7) $$\mathrm{From}\;\mathrm{Equation}\;(3),{\;\mu }_{\mathrm{eff}100}={\;\mu }_{\mathrm{eff}0}-{\alpha I}_{a}^{2}$$

As mentioned earlier, the change in mobility is assumed to be related to the square of the applied loop current because the change in mobility shows better linearity with the square of the applied loop current, as shown in Figure 5b.

Replacing the value of $\mu_{\mathrm{eff}100}$ from Equation (7) into Equation (6) yields

(8) ΔId=|Id100−Id0|= |Id0.αIa2μeff0|

From Figure 6 it is evident that there is a linear relationship between the applied loop current (I_a_) and the magnetic field B generated by the on-chip metal loop, i.e., I_a_ can be replaced by B in Equation (8).

(9) ΔId=|Id100−Id0|=|Id0.α′B2μeff0|

(10) From Equation (9) ΔIdId0.B=α′Bμeff0=S

Note that α′ is a modified proportionality factor. Equation (10) is the definition of the sensitivity of the magnetic sensor designed by a normal-gate single-drain NMOS transistor. Equation (10) reveals that the sensitivity of this kind of a device is directly proportional to the applied magnetic field which is different from the definition of sensitivity of MagFETs. Since the applied magnetic field is of low strength, the difference current tends to increase in a square relationship with B.

From [12], the definition of sensitivity of split-drain MagFETs is

$$S=\frac{\Delta I_{d}}{I_{d}.B}=\mu_{\mathrm{eff}}G\frac{L}{W}$$

where $\Delta I_{d}$ is the difference current between two drain terminals and G is the geometrical correction factor. It is evident that the difference current in MagFETs is mainly directly proportional to the strength of the applied magnetic field. Moreover, $\mu_{\mathrm{eff}}$ in this case is not affected by the Lorentz force [12]. According to the results presented here, mobility can be altered by applying magnetic fields that ensure the change in drain current only due to a change in mobility of the DUT.

**Figure 7:**
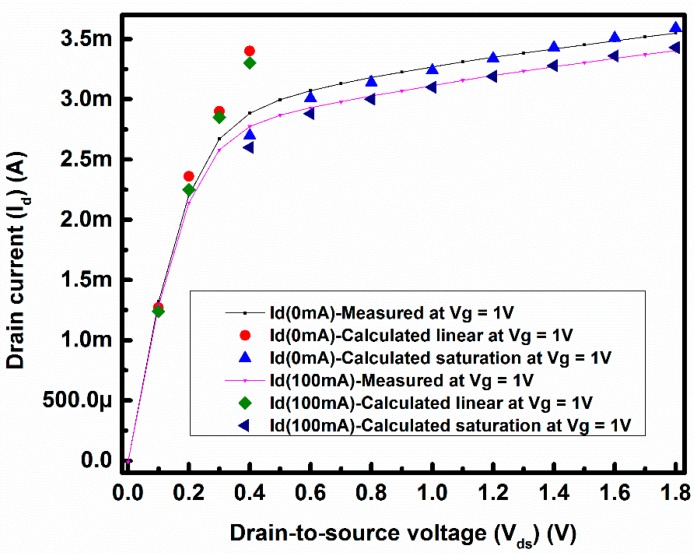
Measured and calculated values of drain current under 0 mA and 100 mA loop current with V_g_ = 1 V.

Using Equations (1) and (2) and with the fitting values of α, θ, η and λ, the drain current with 0 mA and 100 mA loop current were recalculated to verify the validity of the proposed drain current equations. The results are presented in Figure 7. From Figure 7 it can be seen that the calculated drain current agrees well with the measured results except in the transition region between liner and saturation. The discrepancy between liner and saturation regions can be improved by a more accurate determination of the effective drain saturation voltage (V_d,sat_) for the used transistor.

**Figure 8:**
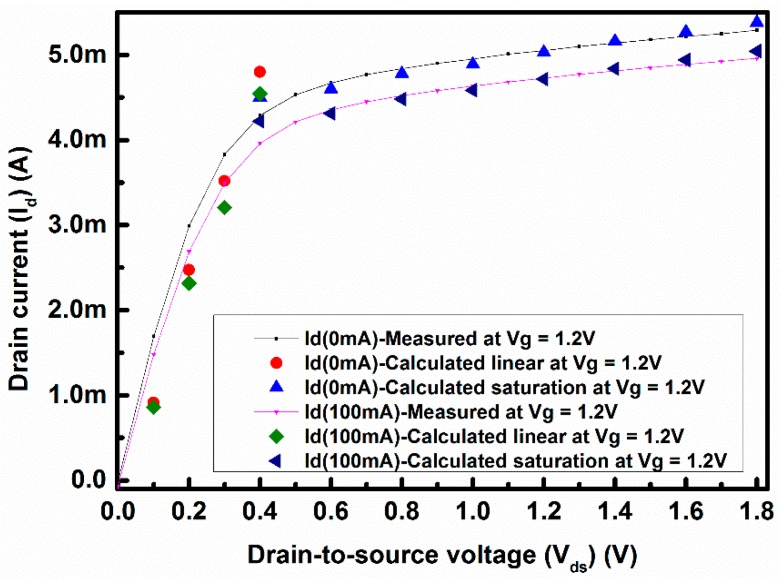
Measured and calculated values of drain current under 0 mA and 100 mA loop current with V_g_ = 1.2 V.

To demonstrate this simple drain current model applicable for other conditions, a set of measured and calculated drain currents without loop current and with a 100 mA loop current is shown in Figure 8 with V_g_ = 1.2 V. The calculated drain current at V_g_ = 1.2 V also follows the measurement results except in the transition region, as in Figure 7. The change in the drain current is a little higher in Figure 8, which may arise from the voltage dependency of α, as mentioned earlier.

To set up for the measurements of the sensor performance, the magnetic field can be applied in two different ways, viz. using the off-chip method or the on-chip method. In the off-chip method, there are many parameters that can affect the measurements, such as both the uniformity and the intensity of the magnetic field or the distance of the field generator from the device surface. In this set-up it is hard to place an external magnetic field generator near the device surface due to the size of the device and to probing difficulties. To avoid these issues, a metal loop was designed on the chip surface to create an on-chip magnetic field. This metal loop is acting as the magnetic field generator for this DUT. The measurements of the magnetic field created by the metal loop are not possible due to the probing issue since the magnetic field sensor cannot reach the chip surface and with distance the magnetic field strength will decrease drastically. Therefore, to estimate the strength of the magnetic field, Equation (11) is used, as mentioned in [7]

(11) B=μ I L22π (x2 + L24) √(x2 + L22)

Using Equation (11), the maximum estimated strength of the applied magnetic field is 1.385 mT under a 100 mA DC current through the square metal loop. Using the value of the applied maximum magnetic field, the sensitivity of this sensor is calculated using Equation (10), and the achieved sensitivity for the gate-to-source voltage (V_gs_) of 1 V and for the drain-to-source voltage (V_ds_) of 1.8 V is around 4.084% (29.6 T^−1^). The calculated sensitivities for V_gs_ of 1.2 V and V_gs_ of 1.6 V are 4.946% and 4.98% at V_ds_ of 1.8 V, respectively. A comparison table (Table 1) has been provided to compare this work with the previous published works and in terms of sensitivity, and it is shown that this sensing device has better sensitivity than others. However, due to the limited width of the metal loop, the applied loop current is limited to 100 mA for reliable measurements. This magnetic sensor (the rectangular single-drain MOSFET) can be able to detect the magnetic field built on-chip ranging from several tens of µT to few mT.

From the discussion it is evident that the mobility of the charged particles in MOSFETs can be altered by changing the magnetoresistance due to the applied Lorentz force through an on-chip metal loop. The change in mobility has demonstrated better linearity with the squared loop current and hence the simple-drain current model includes the square relationship of loop current for the effective mobility. This change in mobility creates a change in I_d_, i.e., in MOSFETs, two different states can be achieved with or without an applied magnetic field. This property of state change can be applicable as a potential magnetic memory element.

## 4. Conclusions

The effect of a low magnetic field, created by an on-chip metal loop, on a rectangular normal MOS transistor has been verified experimentally and theoretically. This created magnetic field has a linear relationship with the applied loop current and hence both the loop current and the generated magnetic field are synonymous. The size of the device has been chosen carefully to suppress the Hall effect and to maximize the magnetoresistance effect by the Lorentz force to achieve maximum sensitivity. The applied Lorentz force changes the magnetoresistance of the charged carriers that creates a change in drain current. This change in I_d_ is ascribed to a change in mobility in the strong inversion region, and a change in mobility of around 4.45% is obtained. Based on these changed values of mobility in the strong inversion region, for the first time as far as we know, a novel set of modified drain current equations of the normal MOS transistor has been proposed. The mobility change shows better linearity with the squared loop current, and the square relationship of loop current is adopted in the drain current model. The proposed model can be used to predict the difference current with the Lorentz force. Furthermore, this device with an on-chip metal loop can be used as a potential magnetic memory element.

## Figures and Tables

**Table 1:** Comparison with the previous works.

Ref.	Dev. Type	Application of Magnetic Field	Device Structure	Maximum Strength of the Applied Magnetic Field (mT)	Sensitivity	Process
[15]	MAGFET	On-chip	Rectangular (dual-drain)	100	2–4%	2.4 µm CMOS
[6]	Pt/MZF/YSZ MOSFET	Off-chip	Rectangular (single-drain)	600	<0.1%	special CMOS
Our work	MOSFET	On-chip	Rectangular (single-drain)	1.385	(1) 4.08% at V_gs_ = 1 V	0.18 µm CMOS
					(2) 4.946% at V_gs_ = 1.2 V	
					(3) 4.98% at V_gs_ = 1.6 V	
